# Identification and Characterization of Novel Matrix-Derived Bioactive Peptides: A Role for Collagenase from Santyl^®^ Ointment in Post-Debridement Wound Healing?

**DOI:** 10.1371/journal.pone.0159598

**Published:** 2016-07-26

**Authors:** Anthony R. Sheets, Tatiana N. Demidova-Rice, Lei Shi, Vincent Ronfard, Komel V. Grover, Ira M. Herman

**Affiliations:** 1 Graduate Program in Cellular & Molecular Physiology, The Sackler School of Graduate Biomedical Sciences, Tufts University, 136 Harrison Ave, Boston, MA, 02111, United States of America; 2 Graduate Program in Cell, Molecular and Developmental Biology, The Sackler School of Graduate Biomedical Sciences, Tufts University, 136 Harrison Ave, Boston, MA, 02111, United States of America; 3 Department of Developmental, Molecular and Chemical Biology, School of Medicine, Tufts University, 136 Harrison Ave, Boston, MA, 02111, United States of America; 4 The Center for Innovations in Wound Healing Research, School of Medicine, Tufts University, 136 Harrison Ave, Boston, MA, 02111, United States of America; 5 Smith & Nephew PLC, 3909 Hulen St., Fort Worth, TX, 76107, United States of America; 6 University of North Texas Health Science Center, College of Pharmacy, 3500 Camp Bowie Blvd., Fort Worth, TX, 76107, United States of America; University of Reading, UNITED KINGDOM

## Abstract

Debridement, the removal of diseased, nonviable tissue, is critical for clinicians to readily assess wound status and prepare the wound bed for advanced therapeutics or downstream active healing. Removing necrotic slough and eschar through surgical or mechanical methods is less specific and may be painful for patients. Enzymatic debridement agents, such as *Clostridial* collagenase, selectively and painlessly degrade devitalized tissue. In addition to its debriding activities, highly-purified *Clostridia*l collagenase actively promotes healing, and our past studies reveal that extracellular matrices digested with this enzyme yield peptides that activate cellular migratory, proliferative and angiogenic responses to injury *in vitro*, and promote wound closure *in vivo*. Intriguingly, while collagenase Santyl^®^ ointment, a sterile preparation containing *Clostridial* collagenases and other non-specific proteases, is a well-accepted enzymatic debridement agent, its role as an active healing entity has never been established. Based on our previous studies of pure *Clostridial* collagenase, we now ask whether the mixture of enzymes contained within Santyl^®^ produces matrix-derived peptides that promote cellular injury responses *in vitro* and stimulate wound closure *in vivo*. Here, we identify novel collagen fragments, along with collagen-associated peptides derived from thrombospondin-1, multimerin-1, fibronectin, TGFβ-induced protein ig-h3 and tenascin-C, generated from Santyl^®^ collagenase-digested human dermal capillary endothelial and fibroblastic matrices, which increase cell proliferation and angiogenic remodeling *in vitro* by 50–100% over controls. Using an established model of impaired healing, we further demonstrate a specific dose of collagenase from Santyl^®^ ointment, as well as the newly-identified and chemically-synthesized ECM-derived peptides significantly increase wound re-epithelialization by 60–100% over saline-treated controls. These results not only confirm and extend our earlier studies using purified collagenase- and matrix-derived peptides to stimulate healing *in vitro* and *in vivo*, but these Santyl^®^-generated, matrix-derived peptides may also represent exciting new opportunities for creating advanced wound healing therapies that are enabled by enzymatic debridement and potentially go beyond debridement.

## Introduction

Acute cutaneous wound healing occurs through a well-orchestrated series of events that culminate in tissue repair and wound closure [1]. Throughout the overlapping phases of wound resolution, the cellular responses to injury are shaped by the microenvironment, especially the extracellular matrix (ECM). In addition to serving as a structural support, the ECM influences cell growth, shape, differentiation, and motility, among other behaviors [2–4]. Matrix-derived signals transduced via integrins and other cell surface receptors regulate platelet activation and clot formation after injury, facilitate monocyte chemotaxis and local macrophage differentiation, and augment phagocytic capacity [3,5]. Additionally, matrix-integrin interactions dictate growth and migration of keratinocytes, fibroblasts, and vascular endothelial cells, control morphogenic processes such as endothelial tube formation during angiogenesis, and determine synthesis of additional matrix components [3,6]. Further, the ECM is a growth factor repository, capable of binding and sequestering pro-healing molecules such as platelet-derived growth factor (PDGF), transforming growth factor-beta (TGFβ), vascular endothelial growth factor (VEGF), and fibroblast growth factor-2 (FGF2), the latter of which possesses bioactivity dependent upon interactions with heparan sulfate proteoglycan (HSPG) [7]. In response to matrix-derived and injury-provoked events, dermal and epidermal cells, alike, further modify their respective extracellular matrix microenvironments, often giving rise to matrix remodeling [3,8]. Such changes in matrix architecture feedback to the cells and provide additional instruction, perpetuating the cycle of dynamic and reciprocal interactions, known as dynamic reciprocity, between cells and their surrounding ECM that are critical for wound repair [3].

Cells continually remodel the ECM composition and functionality through the activity of proteolytic enzymes, including neutrophil and macrophage-synthesized elastase, along with the Cu^2+^ and Zn^2+^-dependent matrix metalloproteinases (MMPs). Indeed, the matrix-remodeling proteases produced by immune effector cells are necessary for removal of devitalized tissue and debris while serving to stimulate microvascular remodeling and angiogenic activation by promoting capillary sprout formation and endothelial cell migration [9,10]. In turn, within the epidermal compartment, ECM remodeling and MMP functionality activate keratinocyte migration and proliferation required for re-epithelialization and wound closure [11]. Along with this, MMP cleavage liberates ECM-bound angiogenic growth factors, and exposes cryptic bioactive matrix domains that are contained within their ‘parent’ ECM components, which may lack active healing activities themselves [7,12]. This enzymatically driven ECM turnover is regulated by the tissue inhibitors of metalloproteinases (TIMPs), which bind to and inactivate MMPs [13]. Whereas organized, acute wound healing is contingent upon the balanced expression of proteases and their inhibitors, chronic wounds, such as those associated with diabetes, vascular insufficiency, and long periods of immobility, are often typified by a pronounced overabundance of ECM-degrading enzymes and remain arrested in a prolonged inflammatory state [14–16]. The severe protease imbalance characteristic of these injuries, concomitant with a marked reduction in protease inhibitors, is hypothesized to cause excessive matrix turnover that disrupts the canon of wound repair [16]. Moreover, excessive proteases, especially neutrophil elastase, may be responsible for destruction of numerous growth factors in chronic wounds, such as PDGF and TGFβ [3,17]. Overall, the loss of ECM-derived signals and trophic factors stemming from MMP-to-TIMP imbalance drastically alters cell-matrix interactions in chronic wounds and impairs post-injury migration, proliferation, and angiogenesis.

The ischemia resulting from angiogenic impairment can lead to wound necrosis [18,19]. This devitalized tissue, largely comprised of denatured collagen, may exacerbate inflammation, increase susceptibility to infection, and presents an impediment to reparative processes. Therefore, the removal of nonviable tissue, slough and eschar through debridement is a critical element of preparing a wound bed to heal [20,21]. Whether achieved through surgical (sharp) intervention, mechanical methods, autolysis, or application of exogenous enzymes, debridement not only allows clinicians to thoroughly assess wound severity, but likely aids in reducing bacterial burden, regulating inflammation, and removing obstructions or barriers to healing. At the same time, outcomes from debridement vary across the spectrum of care delivery and the technical means by which debridement is achieved. However, the mechanisms by which debridement might promote healing are not fully understood. Sharp debridement is a rapid method of clearing tissue, but it is does not selectively remove necrotic material, and results in perioperative site pain. Similarly, wet-to-dry dressings that allow mechanical debridement are even less selective and may also remove viable tissue thus creating a larger wound and more pain for patients. While the use of hydrogel devices and moisture-retaining dressings support immune cell-mediated clearance of necrotic tissue, these processes rely on the body’s own immune system to mount a sufficient response to clear the necrotic tissue requiring several weeks and may require extended treatment periods to achieve complete debridement [21,22]. In contrast, exogenous enzymatic agents that selectively degrade denatured collagen, such as collagenase derived from the bacterium *Clostridium histolyticum*, are painless, non-destructive to healthy tissue, and may remove devitalized tissue more rapidly than the autolysis process [21–23].

In addition to its debriding activities, purified *Clostridial* collagenase stimulates cellular responses to injury. Our previous studies reveal that this enzyme robustly increases proliferation of vascular endothelial cells and keratinocytes, as well as their migration after injury *in vitro* [24,25]. Along with this, *Clostridial* collagenase treatment induces cellular responses to injury *in vivo*, yielding enhanced re-epithelialization and hastened wound closure in Yucatan microswine [25]. In investigating the molecular mechanisms underlying *Clostridial* collagenase-mediated wound healing, our lab identified several collagen- and collagen-associated protein fragments liberated from *Clostridial* collagenase-digested ECM that significantly stimulate endothelial cell proliferation and morphogenesis, keratinocyte migration after injury, and enhance granulation tissue and wound resolution in murine models of impaired healing [26,27]. Together, these data suggest that the pro-healing activities of *Clostridial* collagenase are mediated by the liberation of bioactive matrix fragments that function to promote the migratory, proliferative, and angiogenesis-inducing activities required for wound healing.

Collagenase Santyl^®^ Ointment, a sterile enzyme preparation that contains a mixture of collagenases and non-specific proteases produced by *Clostridium histolyticum* fermentation, is currently the only biologically active enzymatic debridement agent approved by the FDA for clinical use. Enzymatic debridement with collagenase Santyl^®^ has also been associated with improved healing dynamics [28], however the molecular mechanisms linked to these clinical outcomes have neither been characterized nor well understood. Based on our results from experiments with purified *Clostridial* collagenase, we hypothesized that the enzyme combination contained in Santyl^®^ may induce key cellular responses to injury through the production of matrix fragments from specific cleavages made during the debridement process, which stimulates downstream healing activities [25–27]. In the present study, we identify several collagen- and collagen-associated peptides that are liberated due to specific cleavages made when endothelial or fibroblastic ECM are treated with Santyl^®^ collagenase *in vitro*. Collagenase-liberated protein fragments and ECM-derived peptides include the α3 chain of type VI collagen, as well as peptides derived from thrombospondin-1 (TSP-1), multimerin-1 (MMRN-1), and fibronectin, to name several. Herein, we demonstrate some of these peptides promote human dermal capillary endothelial proliferation and microvascular morphogenesis *in vitro*, and stimulate human keratinocyte and dermal fibroblast growth *in vitro*. Finally, using a pre-clinical animal model of impaired healing, we reveal that a specific combinational peptide, created by synthesizing collagenase-liberated peptides adjoined to one another, promotes healing responses *in vivo* by activating granulation tissue formation and wound re-epithelialization.

## Materials and Methods

### Cell culture

Primary adult human dermal microvascular endothelial cells (HMVEC, Lonza Bioscience, Walkersville, MD) were cultured in Endothelial Basal Medium-2 (Lonza) supplemented with 5% fetal bovine serum (FBS) and the growth factors contained within the EGM2-MV kit (Lonza), according to the manufacturer’s instructions. Primary adult human dermal fibroblasts (HDF, Lonza) were grown in Dulbecco’s Modified Eagle’s Medium (DMEM, Life Technologies, Grand Island, NY) supplemented with 10% FBS (Atlanta Biologicals, Atlanta, GA), 1% antibiotic-antimycotic, 1% L-glutamine, and 10mM HEPES (Life Technologies, Grand Island, NY). Primary adult human epidermal keratinocytes (NHEK, Lonza) were cultured as previously described [27]. HMVEC were used at P5-8, HDF were used at P3-8, and NHEK were used at P3-7.

### Matrix Preparation

Extracellular matrices were prepared as previously described [26] and based on the original methods developed [29]. Briefly, HMVEC and HDF at 7–10 days post-confluence were washed three times with Tris-buffered saline (TBS, pH 7.2). Cells were removed using 0.5% sodium deoxycholate buffered with 20 mM Tris-Cl (pH 8.0) containing 150 mM NaCl (pH 7.0) and a protease inhibitor cocktail (P8340, Sigma-Aldrich, St. Louis, MO). Two room temperature detergent treatments were performed, each lasting 15 minutes; subsequently, the decellularized ECM was washed with five times with TBS (1 minute per wash), immediately collected, and used for enzymatic digestion and peptide identification.

### Enzymatic digestion of HMVEC and HDF extracellular matrices

Enzymatic ECM digestion was performed as previously described [26] with the following modifications: lyophilized Santyl^®^ collagenase (the drug substance for Santyl^®^) was obtained from Smith & Nephew PLC (Fort Worth, TX), reconstituted in TBS at 1.0 mg/mL, and stored at -20°C before use for no longer than 1 week. Immediately prior to use, the enzymes were thawed, diluted to 1000 U/mL in TBS containing 2 mM calcium, and added to the decellularized plates for 24 hours at 37°C.

### Peptide identification and synthesis: Liquid Chromatography-Tandem Mass Spectrometry

For peptide identification, soluble and insoluble fractions of HMVEC and HDF matrices digested with Santyl^®^ collagenase were collected into ECM-solubilization buffer containing 20 mM Tris-Cl (pH 8.0), 150 mM NaCl (pH 7.0), 0.1% sodium dodecyl sulfate (SDS), 0.5% sodium deoxycholate, 1% NP-40, supplemented with protease inhibitors (P8340, Sigma-Aldrich). The digested matrices were then mixed with reducing sample buffer containing a final concentration of 2% β-mercaptoethanol (Sigma-Aldrich), heated to 95°C for 5 minutes, separated by SDS-PAGE, and proteins were stained using the SilverQuest Silver Staining Kit (ThermoFisher Scientific, Waltham, MA). Protein bands of interest were excised from the gels, and submitted to Taplin Mass Spectrometry Facility at Harvard Medical School for protein identification. At Taplin, the gel bands were degraded using proteomics-grade trypsin, and subjected to liquid chromatography coupled with tandem mass spectrometry (LC-MS/MS) using Orbitrap mass spectrometers (ThermoFisher Scientific). Over 100 protein fragments were identified in Santyl^®^-digested matrices; of these, 8 peptides derived from HMVEC matrix and 6 peptides derived from HDF matrix, each containing 12–25 amino acids, were selected and submitted to Tufts University Core Facility (TUCF) for synthesis by FastMoc Chemistry, as previously described [26,30,31].

### Cell Proliferation Assays

Synthetic peptides originally identified from HMVEC and HDF-derived extracellular matrices digested with Santyl^®^ collagenase were then tested for their ability to stimulate proliferation of NHEK, HMVEC, and HDF *in vitro*. Cell proliferation assays were performed as previously described [26,27], with the following modifications: cells were plated in their normal growth media at a density of 2,000 cells/well in two 48-well plates; the next day (day 1 post-plating), cells were detached from the culture surface of one plate using trypsin, and baseline cell counts were obtained using a Coulter Counter model Z-II (Beckman Coulter, Inc., Fullerton, CA) according to the manufacturer’s instructions. Cells in the remaining plates were fed with culture media containing 1% BCS for HMVEC, 1% FBS for HDF, or 50% basal keratinocyte growth media (serum-free media with bovine pituitary extract and recombinant human epidermal growth factor) for NHEK. Peptide treatments were administered in basal media on day 1 and day 3 post-plating, at a concentration of 100 nM. Cell counts were performed on day 5 post-plating. Recombinant human FGF2 (R&D Systems, Minneapolis, MN), recombinant human PDGFbb (R&D Systems), and Santyl^®^ collagenase (Smith & Nephew PLC, Fort Worth, TX) were used as positive controls for assays with HMVEC, HDF, and NHEK, respectively. A total of three experiments were performed, and each independent experiment contained three technical replicates for each condition assayed. For each treatment condition, relative proliferation was calculated by normalizing the average cell count obtained from the three technical replicates against the average cell count for control treatments. Mean proliferation, standard deviation, and statistical significance were calculated from the normalized values derived from each independent experiment.

### 2D Angiogenesis/Endothelial Morphogenesis

The morphogenic capacity of the ECM-derived peptides was evaluated as previously described [26,27], with the following modifications. Briefly, growth factor-reduced (GFR) Matrigel (Corning Life Sciences, Tewksbury, MA) was thawed on ice overnight in the cold room, and used the next day. Stock solutions of peptides were prepared in sterile water or 1 M Tris (pH 8.0) and diluted in Matrigel to final concentration of 100 nM. After thorough mixing, peptide-containing GFR Matrigel was placed in wells of 8-well chamber slides (BD Biosciences, San Jose, CA). GFR Matrigel blended with 10 ng/mL (0.6 nM) FGF2 served as a positive control, while GFR Matrigel blended with 1% BCS served as a negative control. All peptide/Matrigel mixing procedures and chamber slide preparations were performed at 4°C to prevent premature matrix polymerization. Subsequently, the chamber slides containing GFR Matrigel mixtures were placed in tissue culture incubators at 37°C for 45–60 minutes to allow for matrix polymerization. Wells were then inoculated with 5X10^4^ HMVEC in DMEM supplemented with 1% BCS +/- FGF2 or the corresponding peptides. Phase contrast microscopy and image analysis of endothelial sprout formation at 5 hours post-plating were performed as described [26]. A total of three experiments were performed, and each independent experiment contained three technical replicates for each condition assayed. Relative endothelial tube formation in each experiment was calculated by normalizing the average sprout length measured for each condition to the average sprout length for control treatments. Mean tube formation, standard deviation, and statistical significance were calculated from the normalized values obtained from each individual experiment.

### In Vivo Studies

#### Ethics statement

This study was performed in strict accordance with the recommendations set forth in the National Institutes of Health Guidelines for the Care and Safety of Laboratory Animals. The Tufts University Institutional Animal Care and Use Committee approved all animal protocols and procedures (Protocol Number: B2012-62). All surgeries were performed under ketamine and xylazine-induced general anesthesia, and Buprenex was administered subcutaneously for perioperative analgesia. Euthanasia was performed by lethal CO_2_ inhalation.

#### Murine model of impaired healing and full thickness excisional wounding

Adult Balb/c mice were obtained from the Jackson Laboratory (Bar Harbor, ME) and housed in the Tufts Division of Laboratory Animal Medicine (DLAM). After acclimation, healing impairments were induced as previously described [27] following procedures approved by the Tufts University IACUC in accordance with NIH guidelines for the care and safety of laboratory animals. Briefly, animals were treated with two doses of pharmaceutical grade cyclophosphamide (CY, Sigma-Aldrich) dissolved in sterile saline, administered via intramuscular injection into the caudal thigh at 4 days (150 mg/kg) and 1 day (100 mg/kg) prior to injury. Subsequently, CY-treated mice underwent IACUC-approved cranial excisional wounding surgeries as previously described [27], with the following modifications. Mouse heads were depilated (Nair, Carter-Wallace, New York, NY) the day before surgery. Mice were anesthetized by an intraperitoneal injection of a ketamine-xylazine cocktail (90 mg/kg ketamine and 10 mg/kg xylazine) for surgical procedures and during all manipulations and treatments. Full-thickness excisional wounding was performed using sterile 4mm-diameter punch biopsy tools, sterile scissors, and sterile forceps. Wounds were dressed with Tegaderm (3M, St. Paul, MN) immediately after injury. Peptides were suspended in autoclaved 3% carboxymethylcellulose (CMC) in PBS at concentrations of 0.1 mg/mL or 1.0 mg/mL and injected beneath the Tegaderm bandage using a 25G needle. CMC alone or CMC containing Santyl^®^ collagenase (1.0 mg/mL) served as negative and positive controls, respectively. All treatments were administered daily for a period of 5 days, beginning on the first day after injury, and dressings were changed as necessary. All treatment groups contained at least 4 mice.

#### Tissue harvesting

Animal tissues were collected at 5 days post-injury as previously described [27]. Briefly, after euthanasia by CO_2_ inhalation, cranial wounds, together with underlying fascia and connective tissue, and approximately 5 mm of intact dermal and epidermal tissues surrounding the wounds were excised. The wounds and adjacent uninjured tissues were then bisected; one half was placed in 10% phosphate buffered formalin (ThermoFisher Scientific) and reserved for staining with hematoxylin and eosin (H&E) and Masson’s trichrome, and the other half was embedded in Tissue-Tek OCT compound (VWR, Radnor, PA), frozen on dry ice, and reserved for immunohistochemistry.

#### Quantitative histological analyses of tissue responses to injury

After fixation for 24 hours, tissues were embedded in paraffin, cut into 5 μm-thick sections, and stained with H&E or Masson’s trichrome. Microscopic imaging of stained sections of cranial excisional wounds and digital photomerging were performed as previously described [27]. To determine the percent wound closure, the total wound width and the linear distance covered by keratinocytes migrating from the edges of the wound were each quantified using the freehand line tool in NIH Image J, and the percent of wound closure was determined by dividing the total linear distance of keratinocyte migration from the wound edge by the total width of each wound.

#### Data analysis and statistics

All *in vitro* and *in vivo* experiments were performed at least three times, and all *in vivo* treatment groups contained at least 4 mice. Mean values were compared for each treatment group, and data are displayed as mean fold change relative to negative controls, +/- standard deviation. Statistical analyses of peptide treatments versus negative controls were performed using an unpaired Student’s T-test, with p-values < 0.05 considered to be statistically significant.

## Results

### Santyl^®^ collagenase releases peptides from endothelial and fibroblast-derived extracellular matrices

Although collagenase Santyl^®^ ointment, which contains a mixture of *Clostridial* collagenases and other *Clostridial* proteases, is a well-accepted enzymatic debridement agent, its status as an active healing entity remains unknown. Since our previous studies indicate that pure *Clostridial* collagenase significantly promotes cell migration and proliferation *in vitro* and stimulates wound healing *in vivo* [25] through liberation of bioactive, matrix-derived peptides [26,27], we aimed to reveal whether Santyl^®^ collagenase, through its proven and selective debridement activity, similarly liberates specific collagen- and collagen-associated peptides that promote cellular responses to injury, including cellular growth and capillary endothelial cell-driven angiogenesis *in vitro*; and, if so, whether such bioactive peptides could further induce wound-healing angiogenesis and re-epithelialization in murine models of impaired healing [27]. Indeed, HMVEC- or HDF-derived ECM digested with Santyl^®^ collagenase yield several distinct protein fragments. To determine the identity of the ECM-derived molecular species released during Santyl^®^ collagenase-mediated ECM digestion, liquid chromatography-tandem mass spectroscopy was performed on select SDS polyacrylamide gel-electrophoresed protein bands of interest and present in the collagenase-digested but not control treated ECM preparations. Linked to these experimental goals, LC-MS/MS [31] was performed on ~25 kDa and ~65 kDa protein bands excised from SDS gels containing electrophoresed HMVEC or HDF ECM extracts, respectively. After LC-MS/MS-based identification of the peptides generated from Santyl^®^-digested ECM, we characterized several, key peptides that contained domains or domain fragments possessing conserved motifs that have been demonstrated to play roles in regulating in cell migration, proliferation and/or angiogenesis, e.g. peptides possessing the N-terminal domain of TSP-1 or the EGF-like Ca^2+^-binding domain, among others [32,33], as shown in Table 1 and Table 2. To these ends, we investigated the bioactivity of 8 unique peptides that originate from TSP-1 (TSN1, 2), MMRN-1 (TSN3-6), and fibronectin (TSN7, 8) and are released from Santyl^®^ collagenase-digested HMVEC matrices. Following Santyl^®^ collagenase-digestion of HDF matrices, several unique peptides are liberated, including those derived from TGFβ-induced protein ig-h3 (TGFBI, TSN11, 12), tenascin-C (TSN14, 15), and the α3 chain of collagen VI (TSN16, 17). In addition, and as we have previously reported [26,27], we have recombined several domains or fragments from a few of the peptides identified, creating ‘combinatorial’ peptides to explore whether such recombined peptides that possess multiple, key signaling domains might be more efficacious in promoting wound healing responses *in vitro* or *in vivo*. Thus, we created combinatorial peptides comprised of individual domains contained within the HMVEC (TSN9, 10) and HDF (TSN13) matrix-derived peptides, or which contain fragments of additional matrix protein domains identified through LC-MS/MS (TSN18, 19).

**Table 1:** Sequence and Origin of Endothelial Extracellular Matrix-Derived Peptides.

Peptide Name	Sequence	Peptide Length	Origin
**TSN1**	NFQGVQNRFVFGTP	14	Thrombospondin-1, Laminin G-like, heparin-binding N-terminal domain
**TSN2**	*MENAELD*VPIQSVFTR	16	Thrombospondin-1, Laminin G-like, heparin-binding N-terminal domain
**TSN3**	NTDNIYPESSC	11	Multimerin-1, EGF-like domain
**TSN4**	**PYLGYVFK**	8	Multimerin-1, C1q domain
**TSN5**	MQTVAQLFKTVSSLSLST	18	Multimerin-1, coiled-coil
**TSN6**	HSPDIQLQKGLTFEPIQIK	19	Multimerin-1, coiled-coil
**TSN7**	STITQPYKTLNNARSP	16	Fibronectin, Fibronectin-type III domain 14, Heparin-binding region 2
**TSN8**	RPGPSPEG*TGQSYNY*	16	Fibronectin, Fibronectin-type I domain 12, Fibrin-binding region 2
**TSN9**	*MENAELD*P**PYLGYVFK**	16	Combination of *TSN2* and **TSN4**
**TSN10**	*TGQSYN*Q*Y*SQR**PYLG**V**YVFK**	20	Combination of *TSN8* and **TSN4**

**Table 2:** Sequence and Origin of Fibroblastic Extracellular Matrix-Derived Peptides.

Peptide Name	Sequence	Peptide Length	Origin
**TSN11**	**LYGQTPLETL**	10	TGFβ-induced protein ig-h3, Fasciclin domain 3
**TSN12**	*ELADSPALEIG*	11	TGFβ-induced protein ig-h3, C-terminal domain
**TSN13**	**LYGQTPLETL***ELADSPALEIG*	21	Combination of **TSN11** and *TSN12*
**TSN14**	VSGNTVEYALPTLE	14	Tenascin C, Fibronectin-type III domain 14
**TSN15**	LDSPTAPTVQSTALTWRP	18	Tenascin C, Fibronectin-type III domain 15
**TSN16**	LDGSAPGPLYTGSALDF	17	Collagen VI α3 chain, vWFA domain 3
**TSN17**	GSEGVRSGRSG	11	Collagen VI α3 chain, vWFA domain 6
**TSN18**	**QP***QPLPSPGVGGK*N	14	Combinatorial Collagen VI α3 chain (**non-helical domain +** *non-helical domain*, located between vWFA domains 6 and 7)
**TSN19**	KYTLNP**VIDAS**	11	Combinatorial Fibronectin-type III domain 14 of Fibronectin (beta-strand region 6 + **beta-strand region 7)**

### Matrix-derived peptides produced during Santyl^®^ proteolysis stimulate cellular proliferation

**Fig 1:**
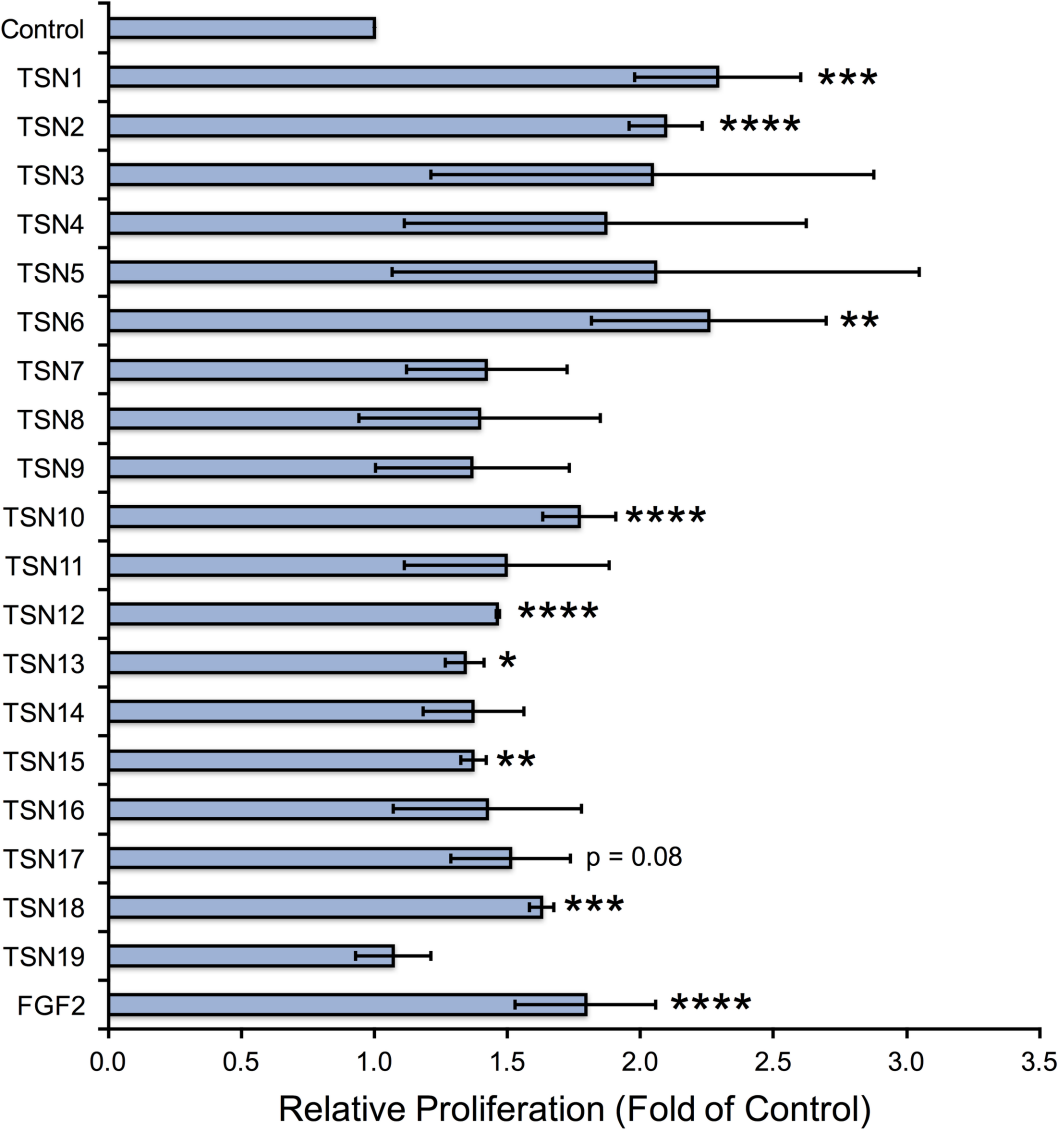
Endothelial and fibroblastic ECM-derived peptides stimulate human dermal microvascular endothelial cell proliferation *in vitro*. Capillary endothelial cells grown in 48-well plates were treated with peptides at 100 nM every other day for a period of 5 days, and cell counts were performed on Day 5 as described in “Methods.” Relative proliferation compared with 1% BCS (control) is shown as fold of control, calculated by normalizing the mean cell count for each treatment to the mean cell count obtained for negative control, and error bars represent standard deviation. * p < 0.05, ** p < 0.01, *** p < 0.005, **** p < 0.001 compared with negative control (unpaired Student’s T-test).

**Fig 2:**
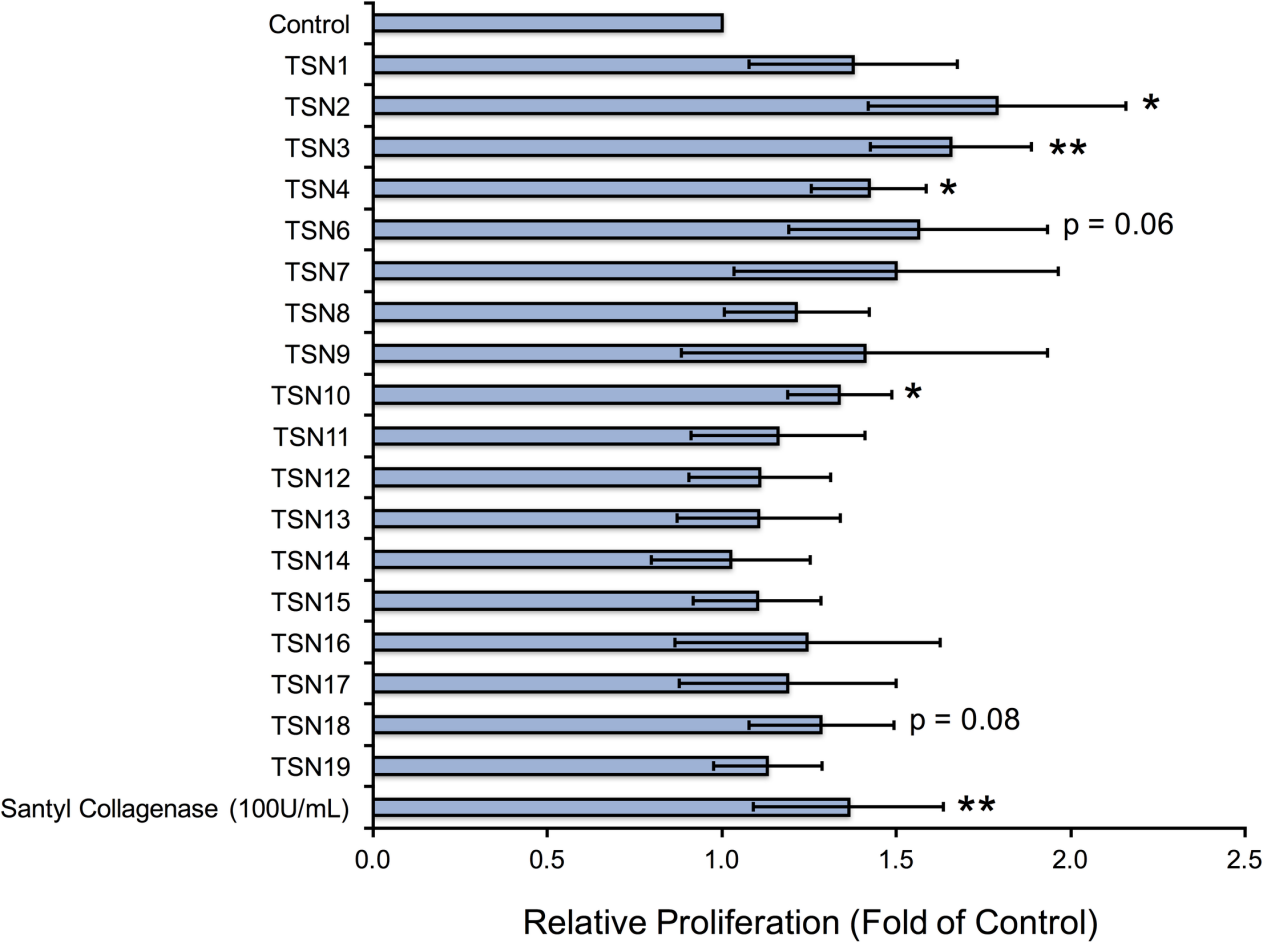
Bioactive endothelial and fibroblastic matrix-derived peptides increase human keratinocyte proliferation *in vitro*. Adult normal human epidermal keratinocytes (NHEK) grown in 48-well plates were treated with peptides at 10 nM every other day for a period of 5 days, and cell counts were performed on Day 5 as described in “Methods.” Relative proliferation compared with 50% basal NHEK media (control) is shown as fold of control, calculated by normalizing the mean cell count for each treatment to the mean cell count obtained for negative control, and error bars represent standard deviation. * p < 0.05, ** p < 0.01, *** p < 0.005, **** p < 0.001 compared with negative control (unpaired Student’s T-test).

**Fig 3:**
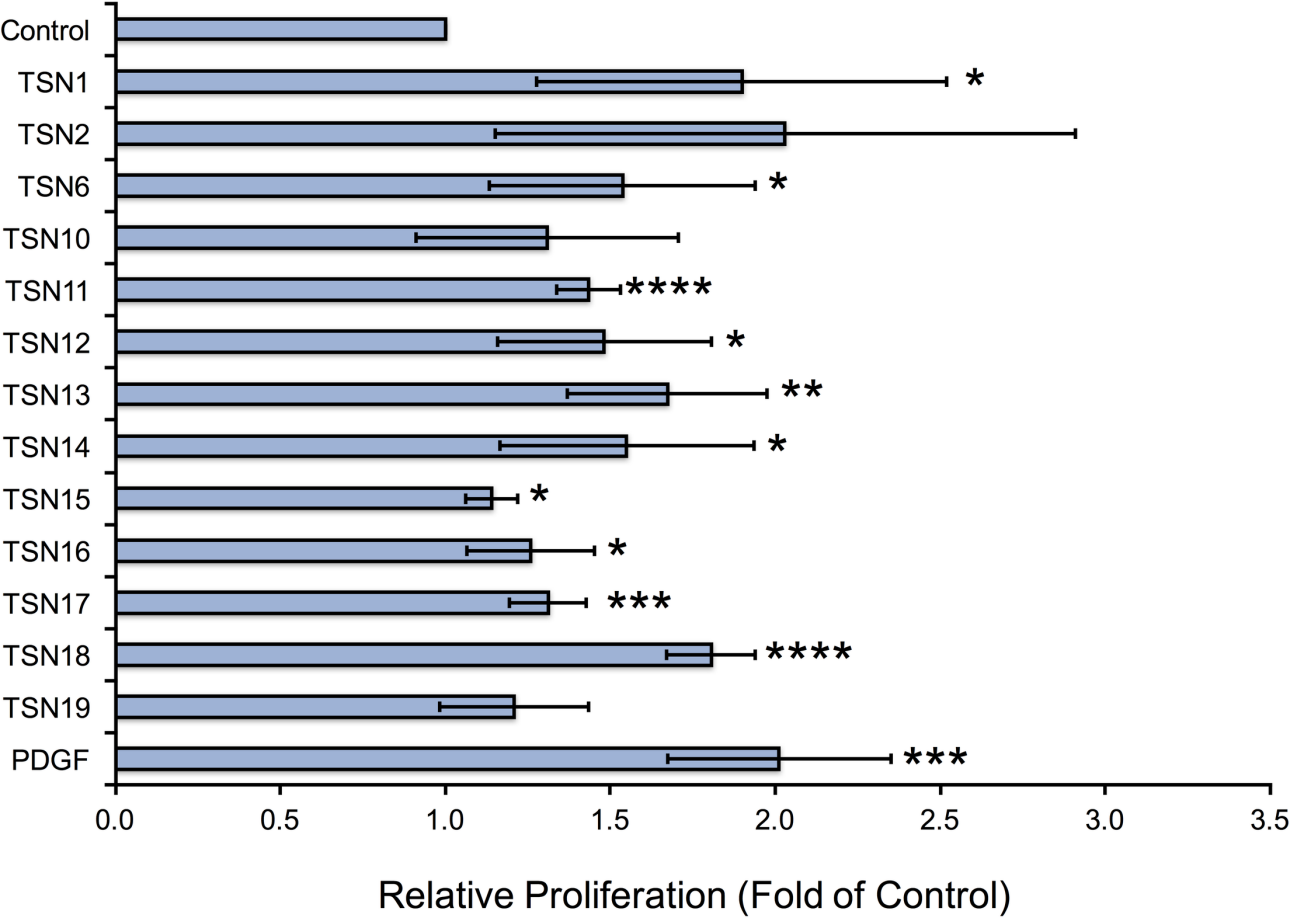
Endothelial and fibroblastic ECM-derived peptides enhance human dermal fibroblast proliferation *in vitro*. Dermal fibroblasts isolated from healthy adult human donors grown in 48-well plates were treated with peptides at 100 nM every other day for a period of 5 days, and cell counts were performed on Day 5 as described in “Methods.” Relative proliferation compared with 1% FBS (control) is shown as fold of control, calculated by normalizing the mean cell count for each treatment to the mean cell count obtained for negative control, and error bars represent standard deviation. * p < 0.05, ** p < 0.01, *** p < 0.005, **** p < 0.001 compared with negative control (unpaired Student’s T-test).

We next asked whether the individual peptides released from Santyl^®^-digested ECM (TSN1-8, 11–12, 14–17), as well as the combinatorial peptides (TSN9, 10, 13, 18, 19), would induce proliferative responses in HMVEC, HDF, and NHEK *in vitro* that would be beneficial for wound healing. Several of the matrix-derived peptides increase endothelial cell proliferation by 50–100% over controls, namely the individual endothelial matrix peptides TSN1, 2, and 6, and the combinatorial peptide TSN10, along with the individual fibroblastic matrix peptides TSN12 and 15, and the combinatorial fibroblastic ECM peptides, TSN13 and TSN18 (Fig 1). Notably, these peptides demonstrate endothelial growth-promoting abilities equivalent to or better than those of physiologically relevant doses of FGF2. The endothelial ECM-derived peptides TSN2, 3, 4 and 6 also significantly elevate keratinocyte proliferation by 40–75%, as does the combinatorial peptide TSN10; while several fibroblastic ECM peptides increase NHEK growth by 20–40% over controls, these changes are not statistically significant (Fig 2). When we treat fibroblasts with TSN1 and 6, proliferation significantly increases by 50–100% over controls; although we observe similar results following treatment with TSN2 and the combinatorial peptide TSN10, these changes are not statistically significant (Fig 3). Additionally, treating HDF with the individual fibroblastic ECM peptides TSN11, 12, 14, 15, 16 and 17 significantly enhances proliferative responses by 20–50%, while treatment with the combinatorial fibroblastic ECM peptides TSN13 and 18 significantly increases cell growth by 50–75% over vehicle-treated controls.

### Santyl^®^-released matrix fragments activate endothelial morphogenesis in vitro

**Fig 4:**
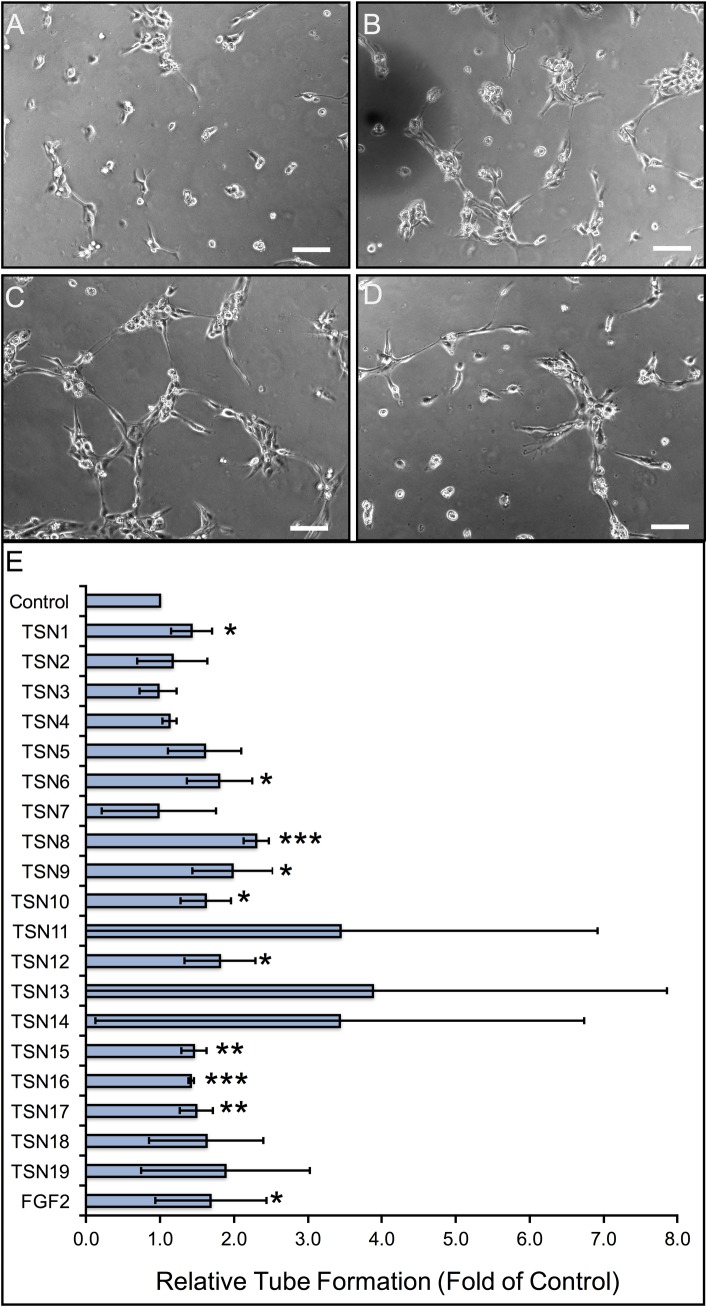
Quantitative analysis of peptide-induced dermal microvascular remodeling *in vitro*. (A-D) Representative photomicrographs of human dermal capillary endothelial cell morphogenesis following treatment with 1% BCS (A), 100 nM TSN1 (B), 100 nM TSN 8 (C), and 100 nM TSN 10 (D). Scale bar represents 100 μm in all panels. (E) The lengths of human dermal capillary endothelial sprouts cultured on growth factor-reduced Matrigel in the presence or absence of 100 nM ECM-derived peptides were measured as described in “Methods” at 5 hours post-plating, with physiologic doses of FGF2 (0.6 nM) serving as positive controls. Data are displayed as fold increase in tube length, relative to 1% BCS-treated negative controls, and error bars represent standard deviation. * p < 0.05, ** p < 0.01, *** p < 0.001, compared with negative control (unpaired Student’s T-test).

As our previous studies indicate that peptides liberated from *Clostridial* collagenase-digested endothelial ECM potently enhance angiogenic activity *in vitro*, we evaluated the morphogenic capacity of the matrix peptides released after Santyl^®^ collagenase treatment using 2D assays of HMVEC seeded on Matrigel. As shown in Fig 4, the individual endothelial ECM peptides TSN1, 6, and 8, as well as the combinatorial endothelial ECM peptides TSN9 and 10 significantly induce angiogenesis *in vitro*, increasing HMVEC tube length by ≥ 50% over serum-treated controls, similar to the results of FGF2 treatment. In addition, the individual fibroblastic ECM peptides TSN12, 15, 16, and 17 significantly stimulate endothelial morphogenesis *in vitro* to a similar extent as FGF2, however the increase in tube formation observed following treatment with the combinatorial fibroblastic ECM peptides TSN13, 18 and 19 is not statistically significant.

### ECM-derived peptides produced by Santyl^®^ collagenase promote tissue repair in vivo

**Fig 5:**
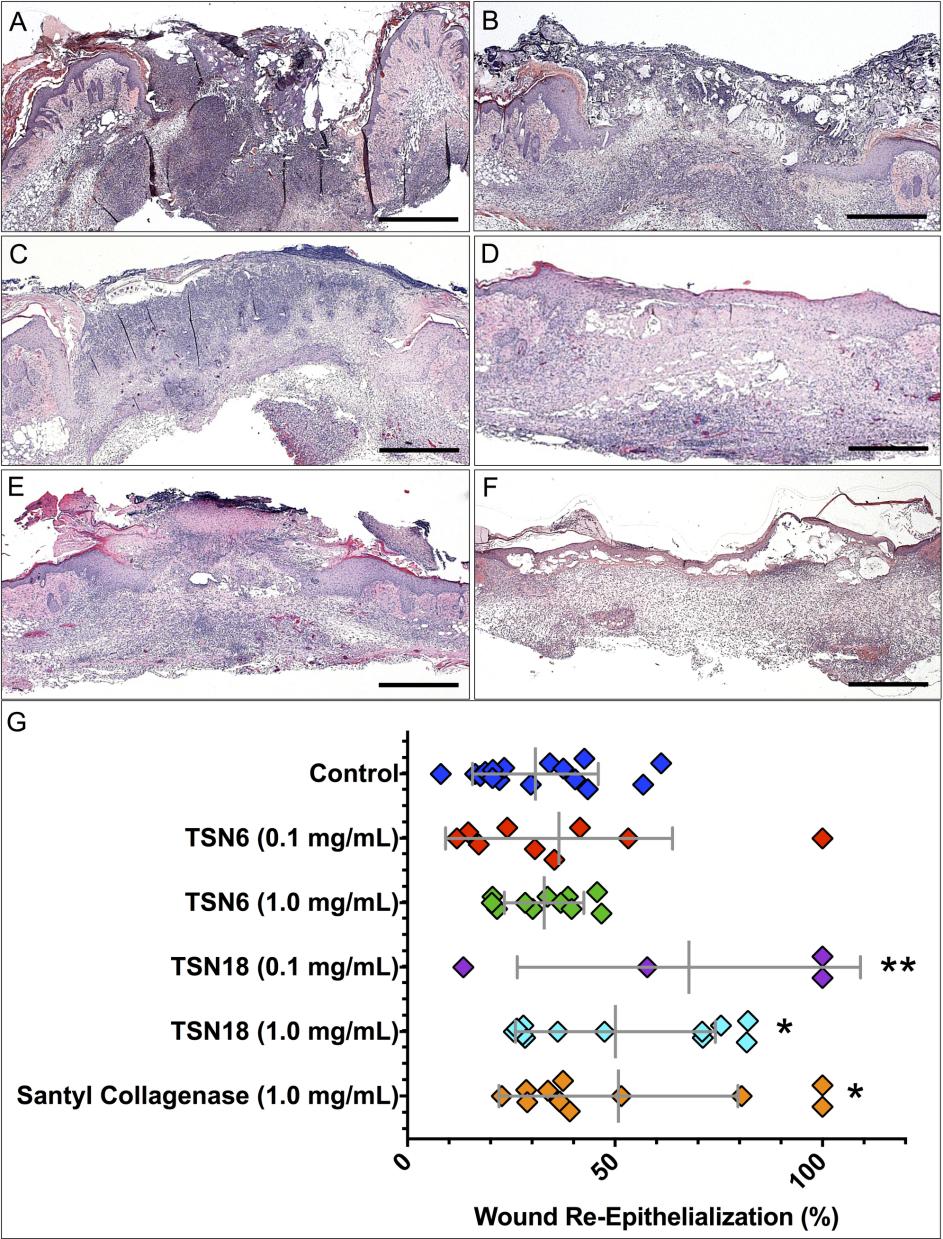
Quantitative evaluation of peptide-dependent wound re-epithelialization. Percent wound closure attributable to re-epithelialization was measured from merged photomicrographs of hematoxylin and eosin or trichrome-stained tissue sections using NIH ImageJ as described in “Methods.” (A-F). Representative photomicrographs of H&E-stained sections of wounds treated with CMC (A), 0.1 mg/mL TSN6 (B), 1.0 mg/mL TSN6 (C), 0.1 mg/mL TSN18 (D), 1.0 mg/mL TSN18 (E), and Santyl^®^ Collagenase (F) are shown. Scale bar represents 1.0 mm in all images. (G). Wounds analyzed for each treatment are plotted as a function of the percent re-epithelialization. Mean percent wound closure is displayed, and error bars represent standard deviation. * p < 0.05, ** p < 0.01, compared with CMC-treated controls.

Having evaluated the peptides for their ability to foster proliferation of endothelial cells, keratinocytes, and fibroblasts, as well as their morphogenic capacity in HMVEC, we next asked whether these peptides would induce wound-healing responses *in vivo*. Based on their overall performance *in vitro* (summarized in Table 3 and Table 4, where peptide-dependent responses in each assay are displayed as mean fold increase relative to control), we chose to test the naturally-occurring endothelial ECM peptide derived from a coiled-coil domain of MMRN-1, TSN6, and the combinatorial fibroblastic ECM peptide created from non-helical domains of type VI collagen, TSN18, to determine the extent to which these peptides foster granulation tissue formation and re-epithelialization in CY-treated, healing-impaired Balb/c mice. The combinatorial fibroblastic ECM peptide TSN18 stimulates dose-dependent improvements in wound re-epithelialization and closure (Fig 5). Whereas wounds treated with CMC alone display ~30% closure, administration of 0.1 mg/mL TSN18 results in ~70% closure, and treatment with 1.0 mg/mL TSN18 yields ~50% closure, similar to the effects of Santyl^®^ collagenase. While 0.1 mg/mL TSN6 induces ~36% wound closure, this change is not significantly different from CMC-treated wounds. Moreover, while the maximal closure we observe with CMC treatment is approximately 60%, several wounds treated with TSN18 display ≥ 70% closure at doses of 0.1 mg/mL and 1.0 mg/mL, some of which are completely epithelialized after peptide treatment (Fig 5).

**Table 3:** Summary of Endothelial ECM-derived peptide bioactivity in vitro. (Numbers in each column represent mean fold increase relative to controls).

**Peptide Name**	**Proliferation**			**Endothelial Morphogenesis**
	Endothelial	Keratinocyte	Fibroblast	
**TSN1**	**2.3**	**1.4**	**1.9**	**1.4**
**TSN2**	**2.1**	**1.8**	**2.0**	**1.2**
**TSN3**	**2.0**	**1.7**	**N/A**	**1.0**
**TSN4**	**1.9**	**1.4**	**N/A**	**1.1**
**TSN5**	**2.1**	**N/A**	**N/A**	**1.6**
**TSN6**	**2.3**	**1.6**	**1.5**	**1.8**
**TSN7**	**1.4**	**1.5**	**N/A**	**1.0**
**TSN8**	**1.4**	**1.2**	**N/A**	**2.3**
**TSN9**	**1.4**	**1.4**	**N/A**	**2.0**
**TSN10**	**1.8**	**1.3**	**1.3**	**1.6**

**Table 4:** Summary of Endothelial ECM-derived peptide bioactivity in vitro. (Numbers in each column represent mean fold increase relative to controls).

**Peptide Name**	**Proliferation**			**Endothelial Morphogenesis**
	Endothelial	Keratinocyte	Fibroblast	
**TSN11**	**1.5**	**1.2**	**1.4**	**3.4**
**TSN12**	**1.5**	**1.1**	**1.5**	**1.8**
**TSN13**	**1.3**	**1.1**	**1.7**	**3.9**
**TSN14**	**1.4**	**1.0**	**1.6**	**3.4**
**TSN15**	**1.4**	**1.1**	**1.1**	**1.5**
**TSN16**	**1.4**	**1.2**	**1.3**	**1.4**
**TSN17**	**1.5**	**1.2**	**1.3**	**1.5**
**TSN18**	**1.6**	**1.3**	**1.8**	**1.6**
**TSN19**	**1.1**	**1.1**	**1.2**	**1.9**

## Discussion

Collagenase Santyl^®^ Ointment is a registered biologic approved by the FDA for enzymatic debridement of necrotic tissue from chronic wounds and severely burned areas. While its clinical application as a debriding agent has been associated with improved healing outcomes, questions remain as to whether it actually stimulates tissue repair as an active healing entity or merely prepares the wound bed for healing via the removal of nonviable eschar. Our earlier and groundbreaking pre-clinical studies indicate that purified *Clostridial* collagenase stimulates the cellular responses to injury *in vitro* and promotes wound closure *in vivo* [24,25]. And, more recently, we discovered and characterized several wound healing peptides, which arise from bio-synthesized ECM digested with purified *Clostridial* collagenase, stimulate wound healing *in vitro* and *in vivo* [26,27]. For these reasons, we postulated that Santyl^®^ collagenase, which contains two collagenases (collagenase G, ~114 kDa and collagenase H, ~110 kDa), a non-specific, neutral metalloproteinase (~35 kDa), and a very limited amount of the cysteine protease, clostripain (~58 kDa), might also contribute to healing via the production of bioactive peptides capable of stimulating cellular and tissue responses to injury.

### Santyl^®^ digestion of endothelial ECM produces several novel bioactive peptides

Our results support several previous studies revealing collagenase stimulation of wound healing [24,25,34], which was later attributed to the proteolytic cleavage of ECM with bacterial collagenase and the liberation of bioactive wound healing peptides derived from collagenous and collagen-associated ECM-derived protein fragments [26,27,35]. Indeed, in this study, treatment of human dermally-derived capillary endothelial extracellular matrices with doses of Santyl^®^ collagenase comparable to those used clinically for debridement yields a collection of soluble protein fragments present in bands that migrate to ~25 kDa when separated by SDS-PAGE. From the proteolysed endothelial ECM, we identified peptides (Table 1) that correspond to the N-terminal laminin G-like heparin-binding domain of TSP-1 (TSN1, 2), EGF-like (TSN3), globular C1q (TSN4) and coiled-coil domains (TSN5, 6) of MMRN-1, and heparin- (TSN7) and fibrin-binding regions (TSN8) of fibronectin. Some of the full-length domains from which the Santyl^®^-released endothelial ECM peptides are derived have already been ascribed roles that may modulate healing responses, as have specific fragments contained therein. For example, laminin G-like domains, which are typically 180–200 amino acids in length, are present in several ECM molecules, and may impact tissue remodeling and angiogenesis dependent upon the number and organization of domains present [36]. Moreover, the full-length N-terminal heparin-binding domain of TSP-1 promotes endothelial cell survival and angiogenesis *in vitro* through two glycosaminoglycan-binding sequences (amino acids 17–35 and 78–94) that interact with syndecan-4 proteoglycan [37,38]. Additionally, amino acids 17–35 of this domain constitute a calreticulin (CRT)-binding sequence that induces endothelial cell and fibroblast focal adhesion disassembly and migration, as well as resistance to anoikis *in vitro*, via signals transduced through a CRT/low-density lipoprotein receptor-related protein-1 (LRP1) co-complex [39,40]. While these events are likely important for wound-healing angiogenesis and granulation tissue formation, intriguingly, *in vivo* implantation of sponges containing plasmids encoding this peptide stimulates the production of a dense, collagen-rich capsule that surrounds the implant, mimicking elements of the foreign body response [41]. At the same time, our data reveal novel functions of peptides and protein domains previously unknown to participate in wound healing, as this is the first study describing bioactivities of peptides corresponding to amino acids 207–221 (TSN1) and 174–190 (TSN2) of the TSP-1 N-terminal heparin-binding domain. Likewise, while the globular C1q-like domain present in MMRN-1 and other elastin microfibrillar interface (EMILIN) proteins has been implicated in cell adhesion, migration and proliferation via interactions with integrins α4®1 and α9®1 [42], to our knowledge this is the first report detailing broad-spectrum bioactivity of fragments derived from the MMRN-1 coiled-coil domains.

### Purified Clostridial collagenase and Santyl^®^ collagenase each liberate unique sets of peptides from endothelial cell matrices

Interestingly, there is little overlap in the endothelial ECM peptides produced by purified *Clostridial* collagenase and those arising from Santyl^®^ collagenase. Indeed, our previous studies identified fragments of fibrillin-1, tenascin X, the α1 chain of type I collagen, and the α3 chain of type IV collagen derived from bovine retinal microvascular endothelial ECM digested with pure *Clostridial* collagenase [26], which we did not detect in our present investigations of human dermal microvascular ECM peptides. Though species and tissue-specific differences may be contributory, human and bovine matrix protein sequence alignments reveal ≥ 85% homology in fibrillin-1 and chains of collagens I, III, IV, V and VI. Thus, while we cannot completely rule out the differences in species or tissue origin, the array of peptides previously identified from bovine endothelial ECM may be specifically linked to the activity of *Clostridial* collagenase, alone, compared to peptides released from human endothelial ECM as a result of the combined enzymatic activities contained within preparations of Santyl^®^. Accordingly, it remains to be determined whether a group of human ECM peptides similar to the previously identified bovine ECM fragments liberated from digestion with purified *Clostridial* collagenase also arise as intermediates during the course of digestion with Santyl^®^ collagenase. For example, it’s possible that the peptides released following ECM treatment with Santyl^®^ collagenase are acted upon by the neutral metalloproteinase and clostripain contained within this enzyme preparation. It is also conceivable that the human ECM peptides identified in the present study are a unique set of matrix fragments produced through the mixed enzymatic activity contained within Santyl^®^. Perhaps the non-overlapping nature stems from the possibility that the specific, collagenase-produced peptides are further cleaved by the non-specific proteases in the mixture, or vice versa, thereby producing the specific bioactive matrix fragments presently detected. Indeed, the distinctions between ECM peptides liberated through the activity of specific proteolytic enzymes cannot be understated, as evidenced from our past studies. For example, when keratinocytes are plated upon a clostripain-digested endothelial ECM cell growth is not stimulated; however, when purified *Clostridial* collagenase is then added to the growth media, these very same cells are markedly stimulated to proliferate [25]. These published findings and our results presented, herein, bolster the notion that unique collections of bioactive peptides may be created through the sequential and/or coordinated activity of selected proteases. Further, it may be of vital importance to combine those peptides previously identified as bioactive wound healing entities [26,27] and those discovered, herein, to learn whether their combined application further enhances wound-healing angiogenesis and re-epithelialization compared to single agent administration.

### Fibroblastic ECM peptides are distinct from endothelial ECM-derived peptides

The peptides extracted from fibroblastic ECM found at ~65 kDa (Table 1) have their origins in fasciclin-1 (TSN11) and N-terminal domains (TSN12) of TGFβ-induced protein ig-h3 (TGFBI), fibronectin-type III domains of tenascin C (TSN14, 15), and von Willebrand Factor A (vWFA) domains of the type VI collagen α3 chain (TSN16, 17). TGFBI is a secreted regulator of cell spreading and adhesion, migration, and morphogenesis during embryonic development and associates with several different proteins in the ECM of fibroblasts and epithelial cells, including biglycan, decorin, and collagen VI [43]. TGFBI mRNA and protein are decreased in fibroblasts derived from chronic wounds, suggesting this protein may be involved in normal wound healing responses [44]. Indeed, the TSN11 peptide derived from the TGFBI fasciclin-1 (FAS1) domain stimulates endothelial migration and morphogenesis *in vitro*, along with fibroblast proliferation, yet the full FAS1 domain has known anti-migratory and anti-angiogenic properties, mediated through disruptions in endothelial integrin αVβ3 and VEGF signaling [45]. These disparate findings suggest unique bioactivies of FAS1 peptides versus the intact domain. At the same, a study by Newman, et al [46] demonstrated a role for fibroblast-derived full-length TGFBI in endothelial lumen formation *in vitro*, in concert with type I collagen and other matrix and matricellular proteins. Though it is unclear whether this latter observation is due to the activity of wholly intact TGFBI per se, it is possible that the mammalian proteases required for ECM remodeling during angiogenesis also cleave TGFBI, liberating morphogenic peptides similar to TSN11/12.

Previous studies have demonstrated *Clostridial* collagenase degrades purified, full-length collagen VI, albeit to a lesser degree than other purified, full-length collagen molecules, including collagens I, III, IV and V [47]. Despite its reduced ability to proteolyze collagen VI, perhaps due to an inhibitory structure at the C-terminus of the α3 chain [47], the inherent differences between the ways in which the enzymes degrade individual, purified matrix proteins vs. those present within a complex, biosynthesized ECM may account for the presence of collagen VI fragments generated from fibroblastic matrices digested with Santyl^®^ collagenase. Additionally, collagen VI may be indirectly associated with other fibrillar collagens and its collagen binding proteins in the biosynthesized fibroblastic ECM, such as collagen I, and the adaptor proteins such as decorin, biglycan and TGFBI [43]. Indeed, the concerted and/or sequential activity of the proteases in Santyl^®^ may act upon the biosynthesized fibrillar and non-fibrillar collagens in the fibroblastic ECM to liberate both TGFBI and collagen VI. In turn, the combination of enzymes in Santyl^®^, including the neutral metalloproteinase and clostripain, may further proteolyze the released TGFBI and collagen VI, thus yielding the peptides identified and characterized in our present study. Our results reveal the growth promoting activities of 2 peptides (TSN16 and TSN17) from collagen VI α3 vWFA domains *in vitro*, along with enhanced wound healing after treatment with TSN18, a combinatorial peptide largely derived from a non-helical region located between vWFA domains 6 and 7. Indeed, mutations in the vWFA domains of collagen VI α3 have been connected with congenital myopathies [48], supporting the trophic role of these motifs; at the same time, the bioactivity of TSN18 points to a novel function of the non-helical domains of this protein. Curiously, collagen VI is also a major component of white adipose ECM, where proteolytic cleavage releases its bioactive C5 domain, known as endotrophin, which upregulates pro-fibrotic and pro-inflammatory genes in white fat and promotes tumor progression [49,50]. These observations, in combination with our data, highlight the diverse bioactivities of collagen VI domains and fragments derived therefrom.

### Santyl^®^ cleaves native and denatured collagen: implications for peptide liberation?

The peptides identified in our study are derived from ECM biosynthesized by healthy cells *in vitro*, which contains molecules in their native states; at the same time, it is notable that the necrotic eschar within non-healing wounds is largely comprised of denatured collagens [51]. In addition to the pre-clinical data contained in the present report, a robust set of pre-clinical and clinical observations strongly support the notion that purified *Clostridial* collagenase, as well as the combination of enzymes present in Santyl^®^, hydrolyze both native and denatured collagen and collagen-associated matrix proteins [25,26,28,47,52,53]. Importantly, despite the differences between the molecules liberated by *Clostridial* collagenases, which consist of several small peptides, and mammalian collagenases (MMPs), which hydrolyze helical collagens into characteristic ¾ and ¼ fragments that are degraded by other proteases [54,55], these enzymes share the ability to digest denatured collagens that have lost their helical organization, as well as the native, helical molecules.

Interestingly, Chung, et al [56] revealed that mammalian collagenase 1 (MMP1) must unwind helical structures prior to peptide bond cleavage, as the intact collagen triple helix is too large to fit in the active site cleft of the enzyme. Indeed, the authors further demonstrated that mutating a key catalytic amino acid residue abolishes MMP1 proteolysis while preserving its ability to unwind the collagen triple helix, rendering collagen susceptible to degradation by otherwise non-collagenolytic enzymes [56]. Moreover, a similar mechanism of action may exist for the *Clostridial* enzyme as well, whereby it uncoils the helical structure before peptide bond hydrolysis [57]. As collagenase-mediated disruptions in the native triple helical winding structure lead to collagen denaturation without impacting the primary amino acid sequence, it is therefore likely that the peptides released from native collagen are highly similar to those liberated from denatured collagen by the activity of either enzyme. In either case (e.g. native vs. denatured collagen), the distinct cleavage patterns of mammalian and *Clostridial* collagenases dictate the specific peptides generated. Based on the combination of enzymes in Santyl^®^ collagenase, it would be of interest to determine whether the loss of helical structure in denatured collagen within eschar impacts the number and/or identities of peptides liberated after Santyl^®^ treatment compared to those we identified from digestion of native, biosynthesized ECM with Santyl^®^.

### Santyl^®^ digestion of endothelial and fibroblastic ECM yields wound healing peptides

Our tests of growth promotion, migration, and endothelial tube formation *in vitro* bring to light pleiotropic effects of endothelial and fibroblastic ECM peptides released after Santyl^®^ collagenase digestion. That some of the endothelial ECM peptides provoke responses in fibroblasts and vice versa may not be so surprising, as the individual contributions of microvascular endothelial and mural cells to their shared basement membrane are known to modulate the behaviors of both cell types during vascular remodeling [58,59]. Similarly, our past studies demonstrate the influence of endothelial matrices on smooth muscle behaviors [29,60], and of fibroblastic ECM components on endothelial shape and migration [61]. Thus, the dynamic reciprocity between cells and the ECM is not strictly homotypic; that is, matrices present in tissues contain products from diverse cell types, which in turn feedback and dictate multiple behaviors in these complex microenvironments.

*In vitro* assays of peptide bioactivity reveal the naturally occurring endothelial ECM peptide, TSN6, and the combinatorial fibroblastic ECM peptide, TSN18 have the greatest stimulatory activity over all parameters and cell types assayed. When we apply these peptides to CY-treated, healing-impaired mice, we observe TSN18 stimulates dose-dependent increases in wound re-epithelialization after full-thickness excisional wounding: 1.0 mg/mL TSN18 produces an effect size similar to treatment with 1.0 mg/mL Santyl^®^ collagenase, each yielding a 60% increase in epithelialization over CMC, while treatment with 0.1 mg/mL TSN18 stimulates a >100% increase of epithelial responses over CMC *in vivo* (Fig 5). Further, 75% of wounds treated with 0.1 mg/mL TSN18 (3/4) and 42% of wounds treated with 1.0 mg/mL TSN18 (5/12) are ≥ 50% epithelialized, whereas 36% of 1.0 mg/mL Santyl^®^ collagenase-treated wounds (4/11) and only 13% of wounds receiving CMC alone (2/16) achieve this mark (Fig 5).

Overall, our *in vivo* studies support the notion that in addition to its debriding activity, Santyl^®^ collagenase improves downstream healing outcomes when applied to full-thickness wounds in mice with healing deficiencies. The enhancements in granulation tissue deposition and wound re-epithelialization may occur through the release of several bioactive ECM peptides, which stimulate human dermal endothelial, keratinocyte, and fibroblast proliferation, as well as microvascular morphogenesis *in vitro*. Importantly, mouse wounds treated with TSN18, a novel ‘combinatorial’ peptide created from fragments of non-helical regions of collagen VI released after digesting fibroblastic ECM with Santyl^®^, display marked improvements in granulation tissue formation and wound re-epithelialization that go well beyond wounds treated under control conditions and are similar to wounds treated with equivalent doses of Santyl^®^ itself. Hence, our future studies will aim to unveil the molecular mechanisms of TSN18-dependent wound healing, while establishing its *in vivo* clinical efficacy.
